# Therapeutic Notes

**Published:** 1899-09-23

**Authors:** 


### THERAPEUTIC NOTES.

### Strychnine in Pneumonia.

In the treatment of catarrhal pneumonia in children Dr. Pepper speaks highly of strychnine when the cough becomes loose and the secretion free. His prescription is as follows:—

Quinine sulph., gr. 24.

Strychnine sulph., gr. $\frac{1}{4}$.

Acid. hydrochlor. dil., fl. dr. $\frac{1}{2}$.

Glycerin., fl. dr. 3.

Liq. pepsinæ ad., fl. oz. 4.

